# ﻿*Xantolisweimingii* (Sapotaceae), a new species from the Yuanjiang River basin, Yunnan, southwest China

**DOI:** 10.3897/phytokeys.246.119516

**Published:** 2024-09-11

**Authors:** Feng Yang, Chao Chen, Qiu-Ping Wang, Jian-Yong Wu, Zhen-Xue Li, Huan-Chong Wang

**Affiliations:** 1 School of Ecology and Environmental Science, Yunnan University, Kunming 650091, China; 2 Key Laboratory of Tropical Forest Ecology, Xishuangbanna Tropical Botanical Garden, Chinese Academy of Sciences, Mengla 666303, Yunnan, China; 3 Yuanjiang Savanna Ecosystem Research Station, Xishuangbanna Tropical Botanical Garden, Chinese Academy of Sciences, Yuanjiang 653300, Yunnan, China; 4 Yuxi Forestry and Grassland Bureau, Yuxi 653100, Yunnan, China; 5 Yunnan Yuanjiang National Nature Reserve Management Bureau, Yuxi 653100, Yunnan, China; 6 Herbarium of Yunnan University, Kunming 650091, Yunnan, China

**Keywords:** Central Yunnan, critically endangered, dry-hot valley, endemism, staminode

## Abstract

*Xantolisweimingii***sp. nov.** (Sapotaceae) is described and illustrated from Yunnan, southwest China. The new species is morphologically most similar to *X.tomentosa* (Roxb.) Raf., but differs from the latter in the ovate or obovate leaves, entirely glabrous corollas, lanceolate, ca. 5 mm long staminodes, fringed at the base. We provided a distribution map and a preliminary conservation assessment for the new species. Additionally, an updated dichotomous key to all known species of *Xantolis* is presented.

## ﻿Introduction

*Xantolis* Raf. (Sapotaceae, Chrysophylloideae) is a small genus of trees and shrubs that comprises approximately 14 species (van Royen 1957; Swenson and Anderberg 2005). Its distribution ranges from the eastern Himalayas to the Philippines in tropical Asia (van Royen 1957; Li 1987; Li and Pennington 1996). This genus is morphologically characterized by having obvious spines, acute anther appendages, lanceolate lobes of calyx and corolla, and aristate staminodes (Swenson and Anderberg 2005). Some members of the genus are of significant economic importance due to their edible fruits and high-quality timber (Li 1987).

The systematic position of *Xantolis* has been controversial. Pennington (1991) classified it as a member of the large tribe Chrysophylleae. Recent studies based on molecular data have demonstrated that *Xantolis* is recovered as a sister to the rest of the subfamily Chrysophylloideae, being a very isolated and poorly understood genus (Anderberg and Swenson 2003; Bartish et al. 2005, 2011; Swenson and Anderberg 2005). Therefore, further extensive sampling is still required to test the monophyly and synapomorphic characters, generic status, and phylogenetic position of *Xantolis* (Triono et al. 2007; Stride et al. 2014; Swenson et al. 2023).

The Yuanjiang River is the mainstream of the upper reaches of the Hong (Red) River, while the Luzhijiang River, situated in central Yunnan in southwest China, is an upper tributary of the Hong River. The rain shadow effect created by the Ailao-Wuliang Mountains and the Yunnan-Guizhou Plateau results in a distinctive hot and dry climate in these valleys, in contrast to most of the surrounding regions (Jin and Ou 2000; Li et al. 2016). The climate in this region is characterized by a dry season (which can be further divided into a cool dry season from November to February and a hot dry season from March to April), with an annual average temperature of 24 °C and a mean annual evaporation capacity of 2700–3800 mm, which is three to six times higher than the mean annual precipitation (600–800 mm). There is also a wet season from May to October, during which 80–90% of the precipitation is concentrated (Jin 2002; Shen et al. 2010; Zhou et al. 2017). The relatively closed environment of the area has led to the formation and retention of a large number of rare and endangered plants and endemic species (Li et al. 2008; Ma 2016). Knowledge of biodiversity in the region seems far from complete, with many new species being described in recent years in different lineages of organisms (Bai et al. 2015; Zhou et al. 2017; Qiao et al. 2018; Yang et al. 2019; Ding et al. 2020; Liu et al. 2022; Ma et al. 2022; Wang et al. 2022; Yang et al. 2022a, 2022b, 2022c; Ma et al. 2023).

The new species of *Xantolis* described here, *X.weimingii* Huan C. Wang & Feng Yang, was first collected in the Luzhijiang Valley in August 2015. During our subsequent fieldwork, we encountered this species several times. However, only sterile or fruiting specimens were collected. In April 2022, the specimen with flowers was finally gathered in Wadie, Yuanjiang County. After a detailed comparison with morphologically similar species, we confirmed its novelty to science and describe it here as *Xantolisweimingii* Huan C. Wang & Feng Yang.

## ﻿Materials and methods

Based on the morphological species concept defined by Cronquist (1978), the morphological studies of the new species were conducted on living plants and specimens coming from the four localities corresponding to the holotype and paratypes. The digital specimen images of similar species available at JSTOR Global Plants (https://plants.jstor.org/), the Smithsonian National Museum of Natural History (https://collections.nmnh.si.edu/search/botany/), and the Global Biodiversity Information Facility (https://www.gbif.org/) were extensively reviewed. Pertinent taxonomic literature (e.g. Clarke 1882; van Royen 1957; Aubréville 1963; Luo 1974; Wu 1977; Li 1987; Luo 1991; Li and Pennington 1996; Pham 1999; Santisuk et al. 2014; Sankara et al. 2019; Turner 2021) were extensively consulted. Measurements were taken using a ruler and a metric vernier caliper under a stereomicroscope (Olympus SZX2, Tokyo, Japan). The dot-distribution map was compiled from all specimens studied and generated with ArcGIS version 10.4 (ESRI, Inc., Redlands, USA). The conservation status was assessed using GeoCAT (online tool available at https://geocat.iucnredlist.org/) (Bachman et al. 2011) to estimate the extent of occurrence (EOO) and the area of occupancy (AOO) of the species, followed by applying the IUCN Red List Categories and Criteria (IUCN 2022) for conservation status assessment. The characters used in the identification key for the congeners mainly followed those of Van Royen (1957) and Li and Pennington (1996).

## ﻿Taxonomy

### ﻿ Xantolis weimingii

Taxon classificationPlantaeEricalesSapotaceae

Huan C. Wang & Feng Yang sp. nov.

3E093BBC-E905-587F-BB95-919946F1F09A

urn:lsid:ipni.org:names:77348184-1

Figs 1 , 2 , 3 , 4

#### Type.

China • Yunnan Province: Yuanjiang County, Wadie village, Luozhi village, near the junction of the Yuanjiang River and Hedihe River, 23°25'51.9"N, 102°18'42.4"E, alt. 1100 m, 14 April 2022, *C. Chen & Z. X. Li YJ19450* (holotype: YUKU 02074716!; isotypes: YUKU!).

**Figure 1.**
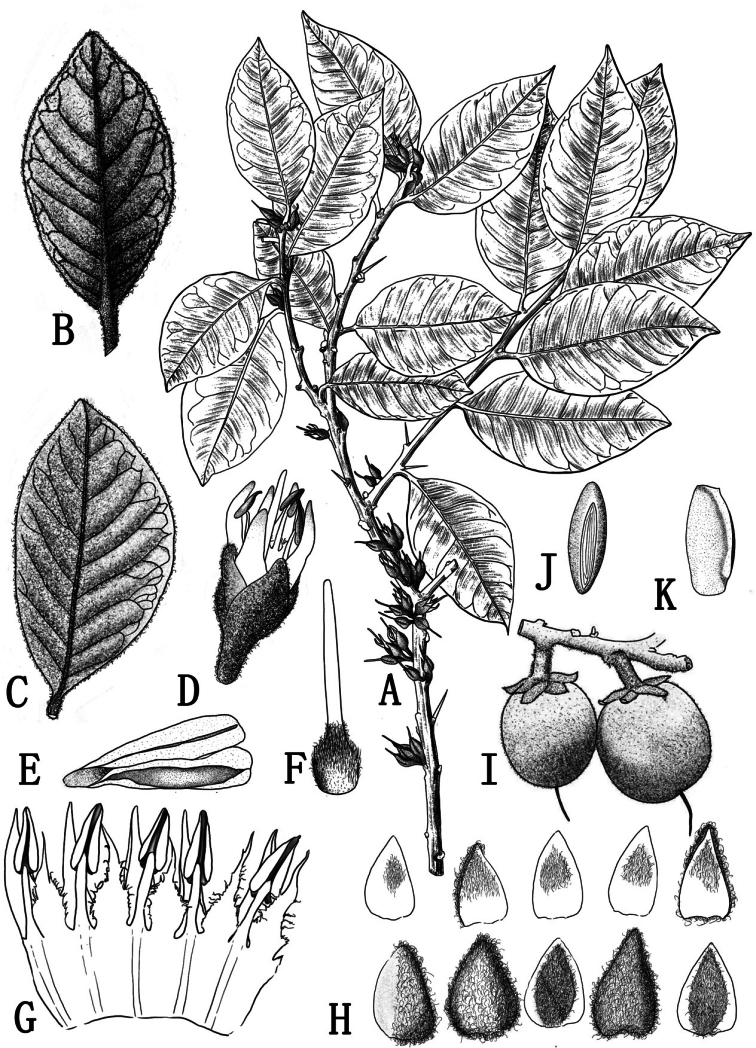
*Xantolisweimingii* sp. nov. (drawn by Qiu-Ping Wang) **A** habit **B** abaxial surface of leaf **C** adaxial surface of leaf **D** flower in blooming **E** anthers **F** pistil **G** corolla dissected to show stamens and staminodes **H** calyx lobes (the upper line is the inner view, the lower line is the outer view) **I** fruits **J** side view of seed to show scar **K** front view of seed.

#### Diagnosis.

*Xantolisweimingii* is most similar to *X.tomentosa* (Roxb.) Raf., but can be easily distinguished by its ovate or obovate (vs. elliptic or elliptic-oblong in *X.tomentosa*) leaves, base broadly cuneate or nearly round (vs. cuneate), apex acute or acuminate (vs. obtuse, short obtusely or acutely acuminate), corollas entirely glabrous (vs. densely hairy at throat), 7.7–9.7 (vs. 4–8) mm long, lanceolate staminodes, ca. 5 (vs. 3–3.5) mm long, apex acuminate into an awn, fringed at the base (vs. broad base, not fringed), glabrous (vs. hairy).

**Figure 2.**
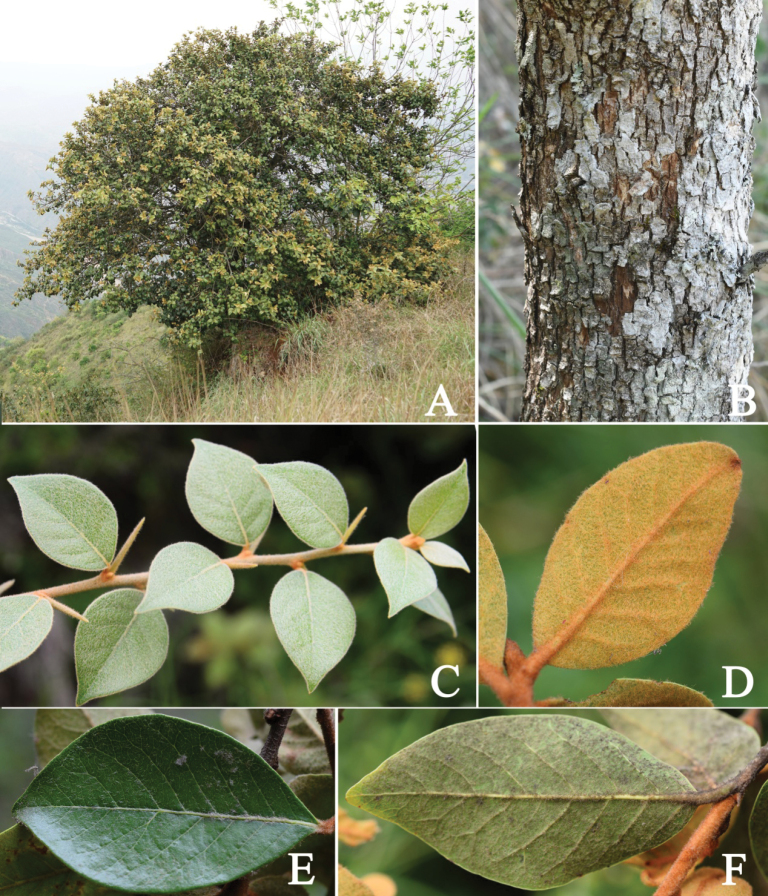
*Xantolisweimingii* sp. nov. **A** habit **B** trunk showing bark texture **C** branchlet **D** abaxial surface of tender leaf **E** adaxial surface of leaf **F** abaxial surface of leaf.

#### Description.

Shrubs or small trees, 2–4 m tall, evergreen, acanthaceous, laticiferous. Bark pale gray, cracked, shallowly and vertically fissured. Branches terete, gray to grayish black; branchlets densely ferruginous arachnoid-lanate, more or less glabrescent when old. Acantha usually axillary, straight, cuspidate, ca. 7 mm long. Petioles 4–8 mm long, with a slight furrow on the adaxial side, densely ferruginous arachnoid-lanate when young, gradually shedding, sparse or glabrescent when old. Leaves ovate to obovate, alternate, leathery, 2.0–8.5 cm long, 1.5–5.0 cm wide, base broadly cuneate or nearly round, apex acute to acuminate, slightly revolute, margin entire, adaxially dark-green, shiny, densely ferruginous arachnoid-lanate when young, glabrescent, abaxially densely ferruginous arachnoid-lanate when young, gradually faded to gray-green sericeous, or to glabrescent when old; midrib flat, obvious adaxially, prominent abaxially, lateral veins 6–9 pairs, arcuate, rising at an angle of 35°–50°, apex bifurcation near the margin, irregularly connected, tertiary and reticulate veins convex abaxially. Flowers in 1–5-flowered clusters in leaf axils or along old branches, pendant. Pedicels stout, terete, 3–4 mm long, densely ferruginous arachnoid-lanate. Calyx cup-shaped, 5-lobed, rarely 4-lobed; sepals imbricate, ovate to triangular, 6–7 mm long, 3.5–4.5 mm wide, apex acute, inside white pubescent on the upper part, outside densely ferruginous arachnoid-lanate. Corolla sympetalous, 5-merous, glabrous, slightly fleshy, tube ca. 4 mm long, lobes lanceolate, 3.7–5.7 mm long, apex acuminate, margin slightly involute, dentate at the base. Stamens 5, adnate to corolla tube at the base, opposite to lobes, filaments white, linear, 2.8–3.5 mm long; anthers sagittate, yellow, ca. 3 mm long, dorsifixed, longitudinal, apex acuminate, base cordate. Staminodes 5, glabrous adnate to corolla tube at the base, alternate to lobes, white, lanceolate, ca. 5 mm long, 1–2 mm wide at the base, apex acuminate into an awn, fringed at the base, glabrous. Ovary ovoid, densely brown pilose; style terete, yellow-green, ca. 8 mm long. Fruits ovoid, oblong or elliptic, with ferruginous arachnoid-lanate hairs, 2.2–4.5 cm long, 1.2–1.5 cm in diam., with persistent calyx, apex sometimes beaked, with persistent style, 1-seeded. Seeds oblong to ellipsoid, slightly compressed, 2–2.5 cm long, ca. 8 mm in diam., both ends truncate, pericarp woody, shiny yellowish brown, scar elliptic, 1.5–2 cm long, ca. 3 mm wide, whitish.

**Figure 3.**
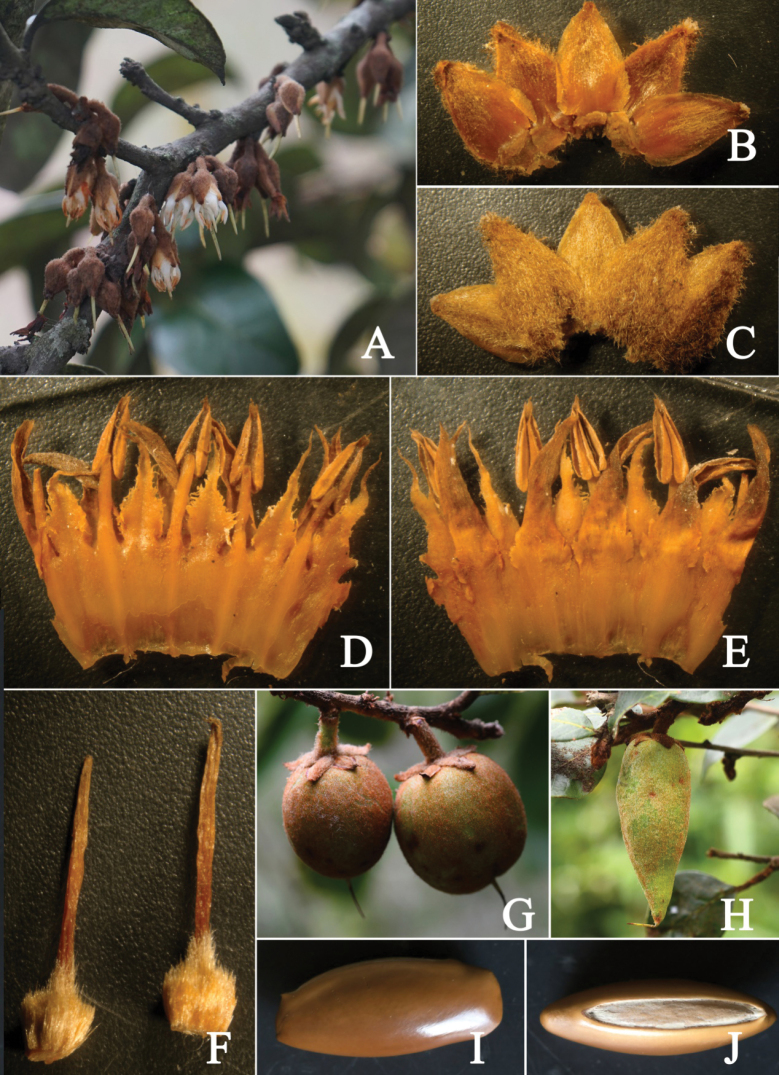
*Xantolisweimingii* sp. nov. **A** flowering branch **B** adaxial view of sepals **C** abaxial view of sepals **D** corolla dissected to show five stamens and five staminodes **E** corolla dissected to show five lobes **F** pistils **G–H** fruits **I** front view of seed **J** side view of seed to show scar.

#### Phenology.

Flowering from April to May, and fruiting from May to October.

**Figure 4.**
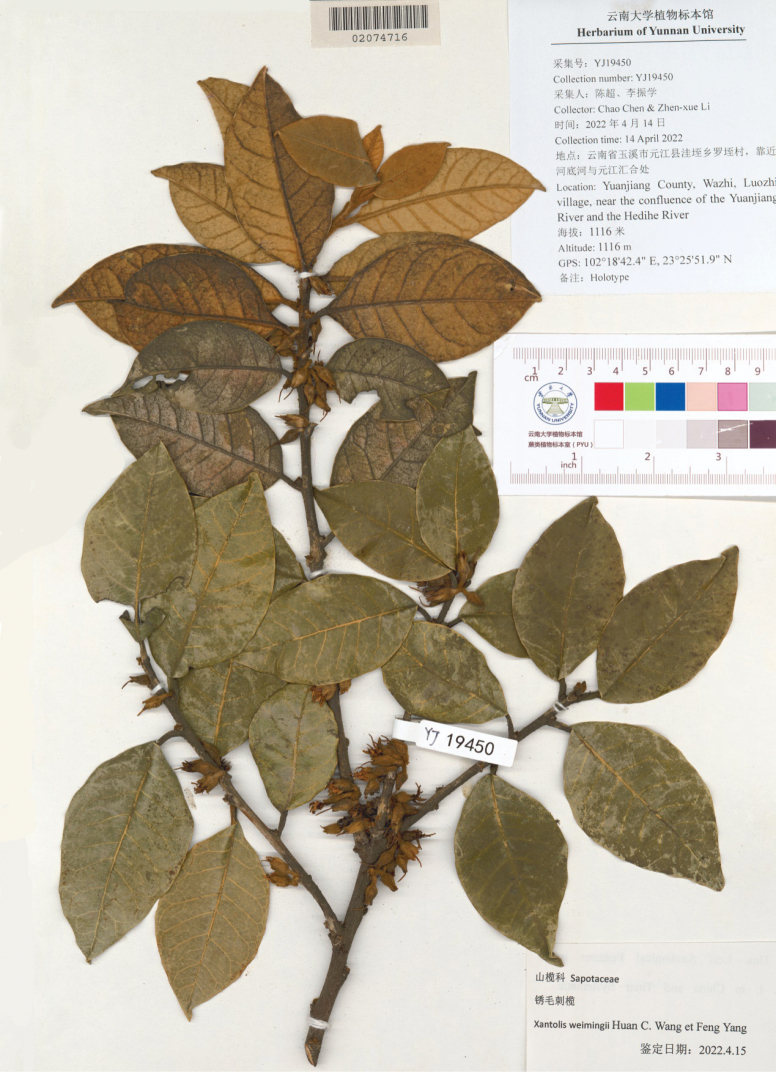
Holotype of *Xantolisweimingii* sp. nov. (YUKU-02074716).

#### Etymology.

The new species is named after Professor Weiming Zhu (朱维明-Wei Ming Chu, 1930–2023), a renowned botanist from Yunnan University, in recognition of his outstanding contributions to the study of China’s flora of Lycophytes and Ferns and to the Herbarium of Yunnan University (Kunming, China).

#### Distribution and habitat.

**Figure 5.**
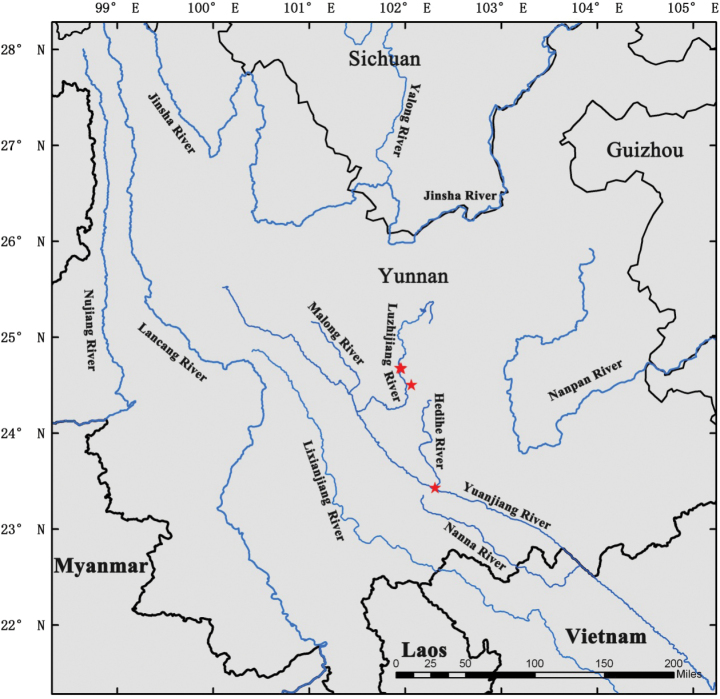
Known geographical distribution of *Xantolisweimingii* (red stars). Based on all known collections.

**Figure 6.**
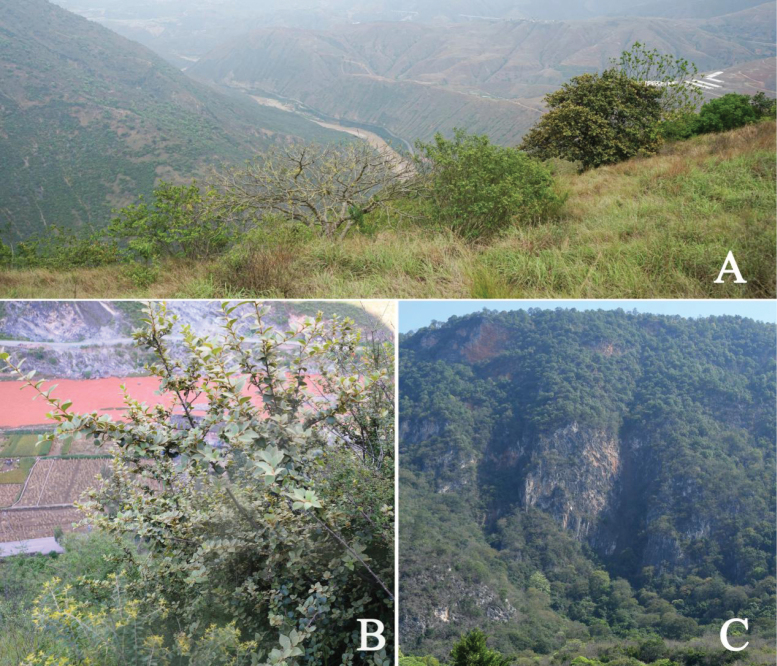
Habitat of *Xantolisweimingii***A** habitat of the Yuanjiang population **B** habitat of the Yimen population **C** habitat of the Eshan population.

*Xantolisweimingii* is a rarely and poorly collected species endemic to the central Yunnan province in southwest China. As of now, it has been discovered in four different sites, all situated in the dry and hot valleys of both the Yuanjiang River and its primary tributary, the Luzhijiang River (Fig. 5). This new species grows in savanna habitats on the mountain slopes at elevations ranging from 1100 to 1400 m (Fig. 6).

#### Preliminary conservation assessment.

*Xantolisweimingii* is at a restricted geographic range, with an estimated extent of occurrence (EOO) of 139.594 km^2^ and an area of occupancy (AOO) of 12 km^2^. Four populations of the new species have been discovered: two of them from the same locality (Yimen County), and one in the Yuanjiang National Nature Reserve. Unfortunately, these populations are typically small, ranging from three to a maximum of eight plants. So far, we have not found any saplings or seedlings in the Yuanjiang and Fawu populations, and we judged that the self-renewal capacity of the wild population of this species is low. The other populations in the Luzhijiang River valley at Yimen County are most threatened. The hillside land here is highly degraded and soil erosion is serious due to mining operations. Furthermore, residents had been harvesting the plant for firewood, resulting in the plant becoming a shrub-like appearance. Therefore, *Xantolisweimingii* is at a high risk of extinction due to a restricted geographic range, fragmented distribution, small population sizes, and fragile living environment. Based on IUCN Red List Categories and Criteria (IUCN 2022), we suggest a Critically Endangered (CR) category for the species.

#### Discussion.

*Xantolisweimingii* can be easily distinguished from its congeners by the following combination of characters: plants densely covered with ferruginous arachnoid-lanate, leaves ovate or obovate, and staminodes fringed at the base. It is most similar to *X.tomentosa* (excluding the synonym *Planchonelladongnaiensis* Pierre ex Dubard), which is widely distributed in Sri Lanka, India, and Myanmar. However, it differs clearly from the latter by having pale gray (vs. light reddish brown in *X.tomentosa*) barks, ovate or obovate (vs. elliptic, elliptic-oblong) leaves, 2–8 (vs. 4–14) cm long, 1.5–5.0 (vs. 2–6) cm wide, base broadly cuneate or nearly round (vs. cuneate), apex acute or acuminate (vs. obtuse or short obtusely or acutely acuminate), 6–9 (vs. 8–l6) pairs lateral veins, 4–8 (vs. 3–20) mm long petioles, 3–4 (vs. 4–7) mm long pedicels, entirely glabrous (vs. throat densely hairy) corollas, 7.7–9.7 (vs. 4–8) mm long, lanceolate (vs. lanceolate-oblong or ovate) lobes, staminodes ca. 5 (vs. 3–3.5) mm long, 1–2 mm wide at the base, apex acuminate into an awn, fringed at the base (vs. broad base, not fringed), glabrous (vs. hairy).

*Xantolisweimingii* is also morphologically similar to *X.cambodiana* (Pierre ex Dubard) P. Royen from Indo-China. Nevertheless, *X.weimingii* differs from *X.cambodiana* in having ovate to obovate (vs. rhomboid-obovate or elliptic, sometimes lanceolate in *X.cambodiana*) leaves, base broadly cuneate or nearly round (vs. tapering towards the base), apex acute to acuminate (vs. obtuse, entire or retuse, sometimes short obtusely acuminate), ovate to triangular (vs. ovate or oblong) sepals, 6–7 (vs. 2.5–4) mm long, 3.5–4.5 (vs. 1–2) mm wide, lanceolate (vs. lanceolate or linear) staminodes, ca. 5 (vs. 2–3) mm long, 1–2 (vs. ca.0.5) mm wide at the base. *Xantolisweimingii* shares similar fruits with *X.assamica* (C.B. Clarke) P. Royen, a species occurring in Assam to Bangladesh, but differs from the latter in its 4–8 (vs. 5–15) mm long petioles, ovate to obovate (vs. ovate, elliptic or broadly lanceolate) leaves, 2.0–8.5 (vs. 6–16.5) cm long, 1.5–5.0 (vs. 2–7) cm wide.

#### Additional specimens examined.

**China • Yunnan**: Yimen County, near Xiaoluzhi village, the west side of Luzhijiang valley, 24°40'46.21"N, 101°56'49"E, 25 September 2015, *H. C. Wang et al. YM241* (YUKU, plant in vegetative period); same location, 27 April 2016, *H. C. Wang et al. YM863* (YUKU, plant in vegetative period); Luzhijiang valley, near Luzhi town, 12 November 2019, *H. C. Wang et al. YM8317* (YUKU, plant in vegetative period); • Luzhijiang valley, near Xiaoluzhi village, Maomao mountain, on the limestone of the dry-hot valley, 24°40'30.9"N, 101°57'37.21"E, elev. 1392.46 m, 25 December 2021, *H. C. Wang et al. YM14630* (YUKU, plant in vegetative period); • Eshan County, Dalongtan, the mountain behind the Fawu village, 24°30'14.17"N, 102°03'46.60"E, alt. 1400 m, 20 August 2015, *H. C. Wang et al. ES173* (YUKU, plant during grain-filling period); • same location, 9 June 2016, *H. C. Wang et al. ES866* (YUKU, plant in late flowering and fruiting period); • same location, 17 September 2017, *H. C. Wang et al. ES2450* (YUKU, plant during grain-filling period); • same location, 27 April 2022, *H. C. Wang et al. YM16402* (YUKU, plant in vegetative period).

### ﻿Identification key to the species of *Xantolis*

**Table:** 

1	Lateral veins numerous, not convex abaxially	**2**
–	Lateral veins few, conspicuously elevated abaxially	**5**
2	Sepals glabrous adaxial, staminodes pubescent adaxial	***X.baranensis***
–	Sepals pubescent adaxial, staminodes glabrous adaxial	**3**
3	Stems sometimes creeping, with numerous spines; leaves suborbicular	***X.maritima***
–	Stems not creeping, sometimes with occasional spines; leaves spatulate, obovate-oblong, obovate or elliptic	**4**
4	Flowers small, corolla 6–9 mm long, lobes lanceolate, 5–6 mm long, ca. 1.5 mm wide, stamens 4–5 mm long, staminodes lanceolate, ca. 3 mm long	***X.parvifolia***
–	Flowers slightly larger, corolla 10–14 mm long, lobes linear, 7–10.5 mm long, 2–3 mm wide, stamens 6–8.5 mm long, staminodes ovate, 4–7.5 mm long	***X.longispinosa***
5	Aspect ratio of mature leaves 1.3–2.5	**6**
–	Aspect ratio of mature leaves 2–4	**11**
6	Pedicels 7–11 mm long	***X.burmanica***
–	Pedicels 3–7 mm long	**7**
7	Staminodes fringed at the base	**8**
–	Staminodes not fringed at the base	**10**
8	Flowers in clusters along 0.7–3 cm long axillary shoots	***X.racemosa***
–	Flowers solitary or in clusters along branchlets	**9**
9	Leaf blades ovate or obovate, apex acute to acuminate; staminodes longer than or equal to stamens	***X.weimingii***
–	Leaf blades rhomboid-obovate or elliptic, apex obtuse, entire or retuse; staminodes shorter than stamens	***X.cambodiana***
10	Leaves spatulate or elliptic, sometimes rhomboid-oblong, 2–3.5 cm long, (0.6–) 1–2 cm wide, base tapering into petioles; secondary nerves 5–10, ascending at an angle of 40°–45°	***X.siamensis***
–	Leaves elliptic-oblong, ovate or obovate, 4–14 cm long, 2–6 cm wide, cuneate at the base, decurrent; secondary nerves 8–16, ascending at an angle of 50°–80°	***X.tomentosa***
11	Leaves 12–22 cm long, 2–7 cm wide, secondary nerves 10–17; pedicels pubescent	**12**
–	Leaves 6–12 cm long, 2.8–5.5 cm wide, secondary nerves 5–13; pedicels glabrous	**15**
12	Sepals ovate, apex subobtuse; corolla lobes 7–9 mm long, 2.5–3.5 mm wide; staminodes 6–7 mm long	***X.hookeri***
–	Sepals lanceolate, apex acute; corolla lobes 3–6 mm long, 1.5–2 mm wide; staminodes 2.5–4 mm long	**13**
13	Leaves ovate, elliptic or broadly lanceolate, 6–16.5 cm long, 2–7 cm wide; secondary veins of leaf 9–15, ascending at an angle of 60°–85°	***X.assamica***
–	Leaves lanceolate, oblanceolate or oblong-lanceolate, 5–18 cm long, 2–5 cm wide; secondary veins of leaf 15–17, ascending at an angle of 40°–55°	**14**
14	Sepals lanceolate to ovate-lanceolate, 4–6 mm long, 1.5–3 mm wide; fruit ferruginous, sericeous to pubescent	***X.stenosepala***
–	Sepals ovate, 3–4 mm long, 2–3 mm wide; fruit subglabrous	**X.stenosepalavar.brevistylis**
15	Corolla lobes fimbriate at the base	***X.shweliensis***
–	Corolla lobes entire	**15**
16	Fruits glabrous; secondary veins of leaf 5–8, ascending at an angle of 35°–55°	***X.boniana***
–	Fruits pubescent; secondary veins of leaf 9–13, ascending at an angle of 50°–65°	**17**
17	Scar of seed as long as the seed, seeds 2–3 cm long	**X.bonianavar.rostrata**
–	Scar of seed 2/3 the length of the seed, seeds up to 2 cm long	**X.bonianavar.pavieana**

## Supplementary Material

XML Treatment for Xantolis weimingii

## ﻿Additional information

### Author ORCIDs

Feng Yang https://orcid.org/0000-0003-2076-0130

Chao Chen https://orcid.org/0000-0001-9533-7340

Qiu-Ping Wang https://orcid.org/0000-0001-7765-4174

Zhen-Xue Li https://orcid.org/0009-0006-9663-4543

Huan-Chong Wang https://orcid.org/0000-0001-8562-8849

### Data availability

All of the data that support the findings of this study are available in the main text.

## References

[B1] Anderberg AA Swenson U (2003) Evolutionary lineages in Sapotaceae (Ericales): A cladistic analysis based on *ndh*F sequence data. International Journal of Plant Sciences 164(5): 763–773. 10.1086/376818

[B2] Aubréville A (1963) Flore du Cambodge, du Laos et du Vietnam vol.3. National Museum of Natural History, Paris, 105 pp.

[B3] Bachman S Moat J Hill AW de la Torre J Scott B (2011) Supporting red list threat assessments with GeoCAT: Geospatial conservation assessment tool (v. beta). In: Smith V Penev L (Eds) e-Infrastructures for data publishing in biodiversity science.ZooKeys 150: 117–126. 10.3897/zookeys.150.2109 PMC3234434 22207809

[B4] Bai L Leong-Skornickova J Xia NH (2015) Taxonomic studies on *Zingiber* (Zingiberaceae) in China II: *Zingibertenuifolium*, a new species from Yunnan, China. Phytotaxa 227(1): 92–98. 10.11646/phytotaxa.227.1.10

[B5] Bartish IV Swenson U Munzinger J Anderberg AA (2005) Phylogenetic relationships among New Caledonian Sapotaceae (Ericales): Molecular evidence for generic polyphyly and repeated dispersal. American Journal of Botany 92(4): 667–673. 10.3732/ajb.92.4.667 21652444

[B6] Bartish IV Antonelli A Richardson JE Swenson U (2011) Vicariance or long-distance dispersal: Historical biogeography of the pantropical subfamily Chrysophylloideae (Sapotaceae). Journal of Biogeography 38(1): 177–190. 10.1111/j.1365-2699.2010.02389.x

[B7] Clarke CB (1882) Sapotaceae A. L. Jussieu In: Hooker JD (Ed.) Flora of British India, Vol.3. Caprifoliaceae to Apocynaceae. L. Reeve and Co., London, 534–549.

[B8] Cronquist A (1978) Once again, what is a species? In: Knutson LV (Ed.) BioSystematics in Agriculture.Alleheld Osmun, Montclair, 3–20.

[B9] Ding HB Yang B Lu XQ Tan YH (2020) *Zingiberporphyrochilum* (zingiberaceae), a new species from Yunnan, China. Annales Botanici Fennici 57(4–6): 197–201. 10.5735/085.057.0401

[B10] IUCN (2022) Guidelines for Using the IUCN Red List Categories and Criteria. Version 15. Prepared by the Standards and Petitions Committee.

[B11] Jin ZZ (2002) Floristic features of dry-hot and dry-warm valleys, Yunnan and Sichuan. Yunnan Science and Technology Press, Kunming, 255 pp.

[B12] Jin ZZ Ou XK (2000) Yuanjiang, Nujiang, Jinshajiang, Lancangjiang: vegetation of dry-hot valley. Yunnan University Press, Kunming, China and Yunnan Science and Technology Press, Kunming, China.

[B13] Li SG (1987) *Xantolis* Raf. In: Li SG (Ed.) Flora Reipublicae Popularis Sinicae, Vol.60(1). Science Press, Beijing, China, 64–67.

[B14] Li SG Pennington TD (1996) Sapotaceae A. L. Jussieu In: Wu ZY Raven PH Hong DY (Eds) Flora of China, Vol.15. Science Press, Beijing, China, and Missouri Botanical Garden Press, St. Louis, Missouri, USA, 205–214.

[B15] Li HT Du F Wang J (2008) Studies on floristics of seed plants in Yuanjiang Nature Reserve in Yunnan province. Journal of Tropical and Subtropical Botany 16: 446–451.

[B16] Li XH Liu YH Liu Y Xu Y Yang Y Shen ZH (2016) Impacts of geographical distances and environmental differences on the beta diversity of plant communities in the dry-hot valley of the Yuanjiang River. Shengwu Duoyangxing 24(4): 399–406. 10.17520/biods.2015245

[B17] Liu JL Li SG Yang F Wang HC (2022) *Indigoferavallicola* (Fabaceae), a new species from Yunnan, southwest China. PhytoKeys 199: 9–16. 10.3897/phytokeys.199.85437 36761874 PMC9848880

[B18] Luo XR (1974) *Xantolis* Raf. In: Chen HY (Ed.) Flora of Hainan, Vol.3. Science Press, Beijing, China, 156–158.

[B19] Luo XR (1991) *Xantolis* Raf. In: Chen FH (Ed.) Flora of Guangdong, Vol.2. Guangdong Science and Technology Press, Guangzhou, China, 352–354.

[B20] Ma XD (2016) Floristic study on the seed plants of Luzhijiang valley in Yunnan, China.

[B21] Ma XD Wang HC He KH Shi JP Shen JY (2022) *Ceropegialuzhiensis*, a new species of Apocynaceae from Yunnan, China. Nordic Journal of Botany 2022(4): e03505. 10.1111/njb.03505

[B22] Ma XD Wang WG Shi JP Shen JY (2023) *Ceropegiaeshanensis*, a new species of Apocynaceae from Yunnan, China. Taiwania 68(1): 75–78.

[B23] Pennington TD (1991) Genera of the Sapotaceae. Royal Botanic Gardens, Kew.

[B24] Pham HH (1999) An Illustrated Flora of Vietnam, Vol. 2. Young Publishing House, Ho Chi Minh City, Vietnam, 638–639.

[B25] Qiao D He ZR Ma XD Wang HC (2018) *Pterygiellaluzhijiangensis* sp. nov. (Orobanchaceae), a new procumbent species from Yunnan, southwest China. Nordic Journal of Botany 36(4): 1–4. 10.1111/njb.01680

[B26] Sankara RK Raja KS Deepak K Arun SR Gopalakrishna BK (2019) Flora of Peninsular India. https://indiaflora-ces.iisc.ac.in/FloraPeninsular/herbsheet.php?id=8883&cat=7 [accessed 7.26.2024]

[B27] Santisuk T Balslev H Newman M Chayamarit K (2014) Flora of Thailand, Vol. 11(4). The Forest Herbarium, Bangkok, 193 pp.

[B28] Shen R Zhang JL He B Li F Zhang ZM Zhou R Ou XK (2010) The structure characteristic and analysis on similarity of grassland community in dry-hot valley of Yuanjiang River. Shengtai Huanjing Xuebao 19(12): 2821–2825.

[B29] Stride G Nylinder S Swenson U (2014) Revisiting the biogeography of *Sideroxylon* (Sapotaceae) and an evaluation of the taxonomic status of *Argania* and *Spiniluma*. Australian Systematic Botany 27(2): 104. 10.1071/SB14010

[B30] Swenson U Anderberg AA (2005) Phylogeny, character evolution, and classification of Sapotaceae (Ericales). Cladistics 21(2): 101–130. 10.1111/j.1096-0031.2005.00056.x 34892862

[B31] Swenson U Lepschi B Lowry PP II Terra‐Araujo MH Santos K Nylinder S Alves‐Araújo A (2023) Reassessment of generic boundaries in Neotropical Chrysophylloideae (Sapotaceae): Eleven reinstated genera and narrowed circumscriptions of Chrysophyllum and Pouteria. Taxon 72(2): 307–359. 10.1002/tax.12894

[B32] Triono T Brown AHD West JG Crisp MD (2007) A phylogeny of *Pouteria* (Sapotaceae) from Malesia and Australasia. Australian Systematic Botany 20(2): 107–118. 10.1071/SB06011

[B33] Turner LM (2021) Heyne, Roth, Roemer and Schultes, and the plant names published in *Novae plantarum species praesertim Indiae orientalis*. Taxon 70(2): 365–428. 10.1002/tax.12449

[B34] van Royen P (1957) Revision of the Sapotaceae of the Malaysian area in a wider sense. VI. *Xantolis* Rafifinesque. Blumea 8: 207–234.

[B35] Wang TT Dang ZY Yang F Wang HC (2022) *Campanulaluzhijiangensis* (Campanulaceae), a new species from Yunnan, southwest China. PhytoKeys 206: 49–59. 10.3897/phytokeys.206.87109 36761272 PMC9848984

[B36] Wu ZY (1977) Flora Yunnanica, Vol. 1. Science Press, Beijing, China, 307–309.

[B37] Yang B Ding HB Fu KC Yuan YK Tan YH (2019) Four new species of Gesneriaceae from Yunnan, southwest China. PhytoKeys 130(2): 183–203. 10.3897/phytokeys.130.34001 31534406 PMC6728316

[B38] Yang F Chen C Ye JY Wu JY Wang HC (2022a) *Breyniahiemalis* (Phyllanthaceae, Phyllantheae), a new species from Yunnan, south-west China. PhytoKeys 206: 75–86. 10.3897/phytokeys.206.85241 36761271 PMC9848962

[B39] Yang F Li PP Liu JL Wang QP Wang HC (2022b) *Breyniapseudorostrata* (Phyllanthaceae), a new species from Yunnan, Southwest China. Phytotaxa 539(2): 210–216. 10.11646/phytotaxa.539.2.8

[B40] Yang F Ye JY Huang QC Wang HC (2022c) *Duhaldealachnocephala* (Asteraceae: Inuleae: Inulinae), a new species from Yunnan, southwest China. Taiwania 67(2): 217–222.

[B41] Zhou Z Gu BJ Sun H Zhu H Tang YH (2017) Molecular phylogenetic analyses of Euphorbiaceae tribe Epiprineae, with the description of a new genus, *Tsaiodendron* gen. nov., from south-western China. Botanical Journal of the Linnean Society 184(2): 167–184. 10.1093/botlinnean/box023

